# Effect of *SORT1*, *APOB* and *APOE* polymorphisms on LDL-C and coronary heart disease in Pakistani subjects and their comparison with Northwick Park Heart Study II

**DOI:** 10.1186/s12944-016-0253-0

**Published:** 2016-04-26

**Authors:** Saleem Ullah Shahid, Shabana ᅟ, Jackie A. Cooper, Katherine E. Beaney, Kawah Li, Abdul Rehman, Stephen Eric Humphries

**Affiliations:** Department of Microbiology and Molecular Genetics, University of the Punjab, Lahore, Pakistan; Centre for Cardiovascular Genetics, British Heart Foundation Laboratories, University College London, London, WC1E6JF UK

**Keywords:** LDL-C, *SORT1*, *APOB*, *APOE*, Gene score

## Abstract

**Background:**

Many SNPs have been identified in genes regulating LDL-C metabolism, but whether their influence is similar in subjects from different ethnicities is unclear. Effect of 4 such SNPs on LDL-C and coronary heart disease (CHD) was examined in Pakistani subjects and was compared with middle aged UK men from Northwick Park Heart Study II (NPHSII).

**Methods:**

One thousand nine hundred sixty-five (1770 non CHD, 195 CHD) UK and 623 (219 non CHD, 404 CHD) Pakistani subjects were enrolled in the study. The SNPs *SORT1* rs646776, *APOB* rs1042031 and *APOE* rs429358, rs7412 were genotyped by TaqMan/KASPar technique and their gene score was calculated. LDL-C was calculated by Friedewald equation, results were analyzed using SPSS.

**Results:**

Allele frequencies were significantly different (*p* = <0.05) between UK and Pakistani subjects. However, the SNPs were associated with LDL-C in both groups. In UK non CHD, UK CHD, Pakistani non CHD and Pakistani CHD respectively, for rs646776, per risk allele increase in LDL-C(mmol/l) was 0.18(0.04), 0.06(0.11), 0.15(0.04) and 0.27(0.06) respectively. For rs1042031, per risk allele increase in LDL-C in four groups was 0.11(0.04), 0.04(0.14), 0.15(0.06) and 0.25(0.09) respectively. For *APOE* genotypes, compared to Ɛ3, each Ɛ2 decreased LDL-C by 0.11(0.06), 0.07(0.15), 0.20(0.08) and 0.38(0.09), while each Ɛ4 increased LDL-C by 0.43(0.06), 0.39(0.21), 0.19(0.11) and 0.39(0.14) respectively. Overall gene score explained a considerable proportion of sample variance in four groups (3.8 %, 1.26 % 13.7 % and 12.3 %). Gene score in both non-CHD groups was significantly lower than CHD subjects.

**Conclusions:**

The SNPs show a dose response association with LDL-C levels and risk of CHD in both populations.

**Electronic supplementary material:**

The online version of this article (doi:10.1186/s12944-016-0253-0) contains supplementary material, which is available to authorized users.

## Background

Coronary heart disease is the leading cause of death in adults all over the world [1, 2]. The prevalence and mortality rate varies in different ethnic groups and people from Indian subcontinent are at greater risk of developing CHD [3, 4]. In Pakistani population, it is estimated that more than 30 % of the people above 45 years of age are affected by CHD [5]. The risk of developing CHD is influenced by both environmental (diet, smoking, lack of exercise) and genetic factors. Identification of common variants in the genese regulating biochemical pathways involved in pathogenesis of the disease may give valuable information.[6]. Blood lipid levels are key modifiable risk factors for CHD and other cardiovascular diseases. Low density lipoprotein cholesterol (LDL-C) is an independent CHD risk factor and LDL-C lowering drugs reduce CHD risk [7, 8]. The biochemical and genetic basis of elevation in blood lipids is not fully understood, but their heritability has been estimated to be at least 50 % [9].

Single nucleotide polymorphisms (SNPs) can be used to examine whether a genetic biomarker is causally linked to a disease risk or not [1]. Most of the SNPs in the genes regulating blood lipids are inherited independently and affect lipid levels quantitatively [10]. In contrast to a large body of evidence available in Caucasians, data on the genetic regulation of lipids in Pakistani population is limited [11]. The allele frequencies of SNPs may show interethnic variations due to different linkage disequilibrium (LD) patterns, genetic drift, gene flow, mutation or admixture. Risk allele frequencies of some SNPs may be more prevalent in specific ethnic groups, or their effects may be modified due to environment or life style changes [12, 13]. This in turn may generate variations in disease outcomes across ethnicities [14]. Studies in non-European people will help to evaluate the true relevance of findings in European people [15, 16]. The *APOE* polymorphisms rs429385 and rs7412 result in three major isoforms of the protein named E2, E3 and E4. Studies have shown that presence of Ɛ4 allele increases concentration of blood lipids particularly LDL-C, while Ɛ2 is LDL-C lowering allele when compared to Ɛ3 [4]. Similarly the effects of SNPs in *APOB* and *SORT1* on LDL-C have been shown by many researchers [17–21]. While there are a large number of studies showing the effect of SNPs in *SORTI*, *APOB* and *APOE* genes with LDL-C levels, their effect needs to be examined and replicated in diverse ethnicities. In this study, we examined the effect of four SNPs on serum LDL-C concentration and CHD. These SNPs are in the genes for *SORT1* (rs646776), *APOB* (rs1042031) and *APOE* (rs429358, rs7412).

The study comprised of two cohorts, one from UK and the other from Pakistan. The objectives of the study were (1) to compare the allele frequencies of the selected SNPs individually and in the form of a combined gene score between UK and Pakistani subjects (2) determining to what extent the SNPs are affecting LDL-C; (3) examining the association of these SNPs with CHD in both populations.

## Methods

### Recruitment of subjects

The Pakistani group consisted of 404 CHD cases collected from hospitals in Lahore covering the whole of the province of Punjab, Pakistan. All subjects were Pakistani and ethnicity was self described by the subject or if both of the grandparents were Pakistani. CHD cases were diagnosed using ECG, cardiac echo, radiologic and troponine T/I data by the cardiologist. All cases were newly diagnosed and were not taking any lipid lowering or anti-hypertensive drugs. Non CHD controls were ethnicity matched healthy individuals without any history of cardiovascular disease and were recruited from general population. The exclusion criteria for CHD cases was the co-existence of any other chronic disease like liver or kidney disease, cancer or any ongoing acute infection. The CHD subjects with obesity were also excluded from the study. In case of controls, the subjects having a family history of early CHD and the obese subjects were excluded. All the subjects were pre-screened for the presence of hepatitis B virus, hepatitis C virus and human immune deficiency virus before starting biochemical and genetic analysis. Seropositive subjects were excluded from the study. The subjects gave a written informed consent and filled in a detailed questionnaire.

The Caucasian samples were from the 2nd Northwick Park Heart Study (NPHSII) described elsewhere [22]. Briefly, NPHSII is a prospective study comprising of middle-aged (50–64 yr) healthy UK men (*n* = 3052) started in 1989. These Subjects were recruited from 9 UK general practices and were followed for a median of 13.5 years for CHD events. The subjects who developed CHD during the follow up period were reffered as CHD while those who remained CHD free were referred as non-CHD. The CHD cases were defined as fatal or non-fatal myocardial infarction or evidence of CHD such as needing angioplasty [23]. For this analysis, only subjects with complete information for all four SNPs were included and the characteristics of this group did not differ significantly from the whole sample set (data not shown).

### Statement of human and animal rights

All procedures followed were in accordance with the ethical standards of the responsible committee on human experimentation (institutional and national) and with the Helsinki Declaration of 1975, revised in 2008. An ethical approval was obtained from the institutional ethical committee for Pakistani samples (PU/27/3-09) and national research ethics service Committee, London Central for UK samples via reference number 14/Lo/1412.

### Statement of informed consent

Informed consent was obtained from all patients for being included in the study.

### Biochemical analysis and DNA extraction

The LDL-C was determined using Friedewald equation in NPHSII [24] and Pakistani samples. DNA was extracted by the salting out method in NPHSII samples. In Pakistani samples, the DNA was extracted with the help of genomic DNA purification kit (Wizard®, USA) using standard protocol. DNA was quantified by nano drop ND-8000 (Labtech, UK).

### Genotyping

For genotyping, samples were first aliquoted into 384 well plates by a robotic liquid handling system, (Biomerk FX, Beckman Coulter). Two florescence based allelic discrimination techniques, TaqMan and KASPar, were used for genotyping the SNPs.

### TaqMan technique

The SNPs rs646776, rs1042031 and rs429358 were genotyped by TaqMan technique (Applied Biosystems). A reaction mixture was prepared for 384 wells plate.The reaction mixture consisted of, 468 μl TaqMan master mix, 23 μl of SNP assay and 410 μl Sigma water. TaqMan master mix consisted of optimized concentrations of a special Taq polymerase enzyme, MgCl_2_, DNTPs, ROX high and Taq buffer. The SNP assay was specific for each SNP and consisted of allele specific primers and probes. The thermal cycler program consisted of a first step at 50 °C for 2 minutes, then initial denaturation/enzyme activation at 95 °C for 10 minutes. This was followed by 40 amplification cycles, each amplification cycle consisted of denaturation at 95 °C for 15 seconds and annealing/extension at 60 °C for 1 min.

### KASPar technique

The SNP rs7412 was genotyped by KASPar (KBiosciences Competitive Allele Specific PCR) technique. The assay mixture for 384 well plate consisted of 900 μl KASPar master mix, 900 μl Sigma water and 26.4 μl of SNP assay. The touchdown thermal cycler program used for KASPar technique consisted of an initial denaturation at 94 °C for 15 minutes, then 10 cycles consisting of 94 °C for 20 sec and annealing/extension temperature reduced from 65 °C to 57 °C lowered by 0.8 °C per cycle. The final round consisted of 26 amplification cycles each amplification cycle consisted of 94 °C for 20 sec and annealing/extension at 57 °C for 60 sec.

After completion of PCR, the results were analysed by florescence resonance energy transmission (FRET) based instrument, ABI PRISM 7900HT (Applied Biosystems). The genotypes were called using sequence detection software (SDS) version 2.0. The genotypes were also confirmed randomly by conventional direct DNA sequencing (source biosciences, UK) to check the accuracy of techniques and the results were always the same.

### Statistical analysis

The results were analysed using statistical package for social sciences (SPSS), IBM version 22.0. The continuous variables like LDL-C and gene score were compared between groups by independent sample *t* test. Hardy Weinberg equilibrium was assessed by a chi squared goodness of fit test. The allele frequencies were compared between different groups by chi squared test. The association of SNPs with LDL-C was estimated by linear regression. The mean LDL-C values against each genotype were calculated by ANOVA. The effect size (β effect) per risk allele is the increase in LDL-C for each additional risk allele held and was calculated by linear regression, denoted by β along with standard error (Se). Differences in regression slope between groups were tested by fitting an interaction term in the regression model. The relationship between gene score, frequency of individuals with a particular gene score and LDL-C was plotted using an excel spread sheet. For all the tests, a *p*-value < 0.05 was considered statistically significant cut-off.

### Calculation of gene score

To combine the information of these four SNPs on LDL-C, an unweighted gene score and a gene score weighted for published effect size on LDL-C in European subjects was calculated. The unweighted gene score was calculated by summing up the number of risk alleles at all the four loci. For the weighted score, the number of risk alleles at that locus was multiplied by the published effect size for the association with LDL-C before summing [25, 26].

## Results

The baseline characters differed between CHD and non CHD subjects in both study groups. The CHD subjects had higher LDL-C concentration, a higher proportion had hypertension and diabetes mellitus and smoking rate was also higher than non CHD subjects. Body mass index (BMI) was significantly higher in NPHSII CHD than non CHD. In Pakistani samples, BMI did not significantly differ between CHD and non CHD subjects. These features were also compared between NPHSII and Pakistani groups. In NPHSII, the proportion of subjects with hypertension and those who were smokers and the mean LDL-C level were significantly higher whereas the proportion of diabetes was lower than in the Pakistani subjects (Table 1).

**Table 1:** Baseline characteristics of NPHSII compared with the Pakistani cohort

Variable	NPHSII samples			Pakistani Samples			P*	P**
	Non CHD	CHD	*p*-value	Non CHD	CHD	*p*-value		
Sample number	1770	195		219	404			
Age (years)	55.9 ± 3.4	56.7 ± 3.5	0.0002	56 ± 10.5	59.1 ± 12.6	0.002	0.87	0.01
Sex								
Males (n)	1770	195		119	238	0.27	*-*	*-*
Females (n)	0	0		100	166			
Diabetes (%)	2.1	5.6	0.006	13.6	64.6	5.1×10^-34^	2.7x10^-19^	5.3x10^-42^
Hypertension (%)	52.6	61.0	0.03	16.4	62.1	8.9x10^-28^	5.5x10^-24^	*0.80*
Smoking (%)	27.8	39.5	0.001	10.5	29.5	7.3x10^-08^	3.5x10^-08^	*0.01*
LDL-C (mmol/l)	3.05 ± 1.01	3.36 ± 0.94	0.00007	2.19 ± 0.44	2.74 ± 0.75	6.5x10^-22^	1.8x10^-34^	3.5x10^-17^
BMI	26.4 ± 3.5	27 ± 3.5	0.019	21.46 ± 9.11	22.46 ± 6.75	0.104		

*NPHSII* Northwick Park heart study II, *CHD* coronary heart disease, P* NPHSII non CHD compared with Pakistani non CHD, p** NPHSII CHD compared with Pakistani CHD, *LDL-C* low density lipoprotein cholesterol, *BMI* body mass index

Continuous variables are expressed in mean ± standard deviation and categorical variables are expressed in numbers with percentage. *P*-value: the level of statistical significance

All the SNPs gave > 95 % genotyping call rates. Hardy Weinberg equilibrium values for both groups are shown (Additional file 1: Table S1). For all the SNPS, risk allele frequencies (RAFs) were significantly different (*p* < 0.05) between the NPHSII and Pakistani groups. The RAF of rs1042031 was lower while those for rs646776, rs429358 and rs412 were higher in NPHSII subjects than Pakistani subjects. In Pakistani group, the the RAF of rs1042031 was significantly higher in the CHD than non CHD (0.92 vs 0.87, *p* = 0.007), while those for rs646776, rs429358 and rs412, the RAFs did not not significantly differ between CHD and non CHD groups. In NPHSII samples, the RAFs did not differ significantly between CHD and non CHD for either of the SNPs studied (Additional file 2: Table S2).

### Effect of SNPs on LDL-C levels

The SNPs were associated with LDL-C in both study groups. The risk alleles of all the four SNPs increased LDL-C quantitatively. The distribution of LDL-C along 3 different genotypes of the SNP, the effect size of risk allele of each SNP (β) the proportion of sample variance (R^2^), and *p* values are given in Table 2. For rs646776, the common allele (A) was the risk allele and β in non CHD subjects from both groups was comparable but a higher value was observed in Pakistani CHD subjects. Similarly for rs1042031, the effect size in Pakistani CHD was higher than NPHSII and Pakistani non CHD. For the *APOE* polymorphisms, rs429358 and rs7412, isoforms Ɛ2, Ɛ3 and Ɛ4 were examined separately. When compared with Ɛ3, Ɛ2 lowered LDL-C while Ɛ4 raised LDL-C in all the study groups. Overall the effect size was significant for all the SNPs in all subjects except NPHSII CHD group. The effect sizes were almost same in healthy UK and Pakistani people but a bigger effect was always observed in Pakistani CHD group (Table 2).

**Table 2:** Mean ± SD LDL-C levels in 3 different genotypes of the SNPs studied

Gene	SNP	Genotype	NPHSII non CHD	NPHSII CHD	Pakistani non CHD	Pakistani CHD
*SORT1*	rs646776	GG	2.75 ± 1.00	2.93 ± 1.08	1.98 ± 0.31	2.42 ± 0.60
		AG	2.95 ± 1.00	3.49 ± 0.86	2.11 ± 0.55	2.57 ± 0.79
		AA	3.13 ± 1.01	3.34 ± 0.95	2.28 ± 0.34	2.88 ± 0.70
		β(se)	0.18(0.04)	0.06(0.11)	0.15(0.04)	0.27(0.06)
		R^2^	1.1 %	0.1 %	5.5 %	4.9 %
		*p*-value	8.7x10^-6^	0.61	0.002	7.4 × 10^-6^
*APOB*	rs1042031	AA	2.93 ± 0.99	2.40 ± 0	1.67 ± 0.12	2.02 ± 0.09
		AG	2.98 ± 0.97	3.36 ± 0.89	2.10 ± 0.42	2.55 ± 0.39
		GG	3.10 ± 1.03	3.36 ± 0.97	2.22 ± 0.44	2.78 ± 0.79
		β (se)	0.11(0.04)	0.04(0.14)	0.15(0.06)	0.25(0.09)
		R^2^	0.3 %	0.04 %	2.6 %	1.7 %
		*p*-value	0.02	0.79	0.03	0.03
*APOE*	rs429358 and rs7412	Ɛ2Ɛ2/Ɛ2Ɛ3/Ɛ2Ɛ4	2.67 ± 1.07	3.00 ± 0.86	1.97 ± 0.34	2.30 ± 0.47
		Ɛ3Ɛ3	3.10 ± 0.98	3.39 ± 0.93	2.17 ± 0.45	2.69 ± 0.71
		Ɛ3Ɛ4/Ɛ4Ɛ4	3.22 ± 0.99	3.45 ± 0.97	2.37 ± 0.35	3.07 ± 0.84
		β(se)	0.26(0.04)	0.19(0.11)	0.20(0.06)	0.38(0.07)
		R^2^	2.7 %	1.6 %	5.2 %	7.0 %
		*p-*value	2.6×10^-12^	0.08	0.003	7.5 × 10^-8^
		Ɛ3Ɛ3 vs. Ɛ4Ɛ4 β(se)	0.43(0.06)	0.39(0.21)	0.19(0.11)	0.39(0.14)
		*p-*value	2.1×10^-12^	0.06	0.07	0.004
		Ɛ3Ɛ3 vs. Ɛ2Ɛ2 β (se)	-0.11(0.06)	-0.07 (0.15)	-0.20 (0.08)	-0.38(0.09)
		*p-*value	0.05	0.67	0.009	0.00004

β (se): beta effect which is increase or decrease (mmol/L) in LDL-C per risk allele along with standard error of mean. R^2^ = proportion of sample variance in LDL-C

### Gene score analysis

**Fig. 1:**
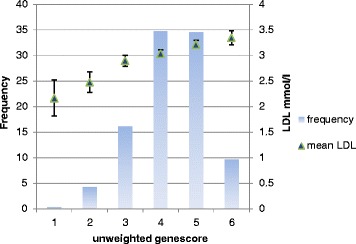
The relation between gene score, frequencies and LDL-C in the NPHSII. Along X-axis is unweighted gene score in NPHSII subjects; along Y-axis on the left is the frequency of the subjects having a specific gene score and right hand side Y-axis shows mean ± LDL-C in a group of subjects with s a specific gene score value

**Fig. 2:**
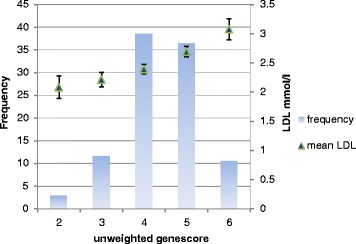
The relation between gene score, frequencies and LDL-C in Pakistani samples. The figure shows the relationship between gene score, LDL-C and frequency of the individuals with a specific gene score and mean ± SD LDL-C. It is clear that by increasing the gene score value, the frequency of subjects with higher LDL-C levels also increase

Individually, the risk allele frequencies were not significantly higher in CHD than non-CHD except rs1042031 in Pakistani samples. However, collectively the unweighted gene score of the four SNPs was significantly higher in CHD than non CHD of both study groups (Table 3, Additional file 3: Figure S1). The effect of gene score on LDL-C was also observed. The effect size of unweighted gene score in NPHSII non CHD was similar to the Pakistani non CHD (0.19 vs 0.17, *p* = 0.82). However, a bigger effect in Pakistani CHD cases (0.29 ± 0.04) was observed than other groups (Table 4). Mean LDL-C values in groups of subjects with different number of LDL-C raising alleles (unweighted gene score) in the NPHSII and Pakistani group is shown in Figs. 1 and 2 respectively.

**Table 3:** Mean gene score ± SD in NPHSII and Pakistani subjects

Gene score	NPHSII samples			Pakistani samples			P*	P**
	Non CHD	CHD	*p*-value	Non CHD	CHD	*p*-value		
Unweighted.	4.26 ± 1.02	4.42 ± 0.98	0.05	4.28 ± 0.94	4.47 ± 0.91	0.02	0.77	0.52
Weighted	12.77 ± 7.99	14.17 ± 7.09	0.02	13.77 ± 6.19	14.5 ± 6.24	0.13	0.07	0.48

P* NPHSII non CHD compared with Pakistani non CHD, p** NPHSII CHD compared with Pakistani CHD

**Table 4:** Correlation of gene score with LDL-C in both groups

Gene score		NPHSII non CHD	NPHSII CHD	Pakistani non CHD	Pakistani CHD
Unweighted	Correlation	r = 0.19	r = 0.11	r = 0.37	r = 0.35
	*p-*value	1.4 × 10^-16^	0.12	1.6× 10^-8^	4.1× 10^-13^
	▫β(se)	0.19(0.02)	0.11(0.07)	0.17(0.03)	0.29(0.04)
	R^2^	3.79 %	1.26 %	13.70 %	12.3 %
Weighted	Correlation	R = 0.23	r = 0.15	r = 0.33	r = 0.34
	*p-*value	1.4x10^-22^	0.04	5.8 × 10^-7^	9.8x10^-13^
	▪β(se)	0.22(0.02)	0.15(0.07)	0.18(0.03)	0.31(0.04)
	R^2^	5.27 %	2.16 %	10.88 %	11.89 %

*r* the correlation coefficient, ▫β is the increase in LDL-C per risk allele of unweighted gene score, ▪β is the increase in LDL per SD of weighted gene score, *R* ^2^ the proportion of sample variance

To observe the impact of increasing number of risk alleles in groups with different baseline LDL-C values, we grouped the individuals according to the number of risk alleles they had. The difference in mean LDL-C levels between those with 6 risk alleles versus those having 2 risk alleles was similar in NPHSII non CHD, Pakistani non CHD and Pakistani CHD group (Additional file 4: Table S3).

## Discussion

In this study, we have analyzed the effect of four SNPs in three genes that were known to have their influence on LDL-C metabolism. We have examined their effect in a Pakistani CHD case control study and compared it with the participants of NPHSII study from UK.. The aim was to compare the allele frequencies and LDL-C raising effect in two groups and to estimate the effect of these SNPs on risk of developing CHD. Although many studies have demonstrated a robust association of these SNPs with LDL-C levels and CHD in different ethnicities, the present study extends this to the subjects from Pakistan.

The difference in allele frequency between European and Asian subjects is not surprising and such differences have been reported for other genes also [11, 14]. We have also recently reported the difference in allele frequencies of 19 CHD related loci between Pakstani and UK people [27]. However, the effect on LDL-C will only be of similar magnitude if either the SNP itself is functional, or if non-functional, the SNP is in strong LD with the functional SNP in both populations studied. The similar effects on LDL-C observed here is compatible with either of these possibilities. However, Pakistani group also included females whereas in NPHSII, only males were included. It has been reported that risk of coronary events increases with increase in LDL-C in postmenopausal females [28] and in females on hormone replacement therapy [29]. The data on these parameters were not recordered for current study and if included in future, it may provide valuable information.

The per allele effects of individual SNPs and gene score on LDL-C did not differ significantly between UK and Pakistani healthy subjects, however the R^2^ (proportion of sample variance) values were considerably higher in the Pakistani samples, indicating a better fit for the models. The UK subjects showed greater variability in LDL-C distribution with a standard deviation of 1.01 compared to 0.44 in the Pakistani controls, suggesting that environmental factors may be having more influence in this population, leading to a smaller proportion of the variance being explained by the genetic component. The non-genetic factors like high rate of urbanization, exposure to tobacco smoke and industrial pollutants, alcohol intake and sedentary life style may be contributing to a greater LDL-C variability in UK people.

The SNP, rs646776 is in complete LD with rs599839 which is a GWAS hit for LDL-C levels [25] and is associated with the expression of *CELSR2*, *PCSR2* and also with *SORT1* which is a gene located in an adjacent LD block, encoding the protein sortilin. *SORT1* expression level is inversely proportional to the circulating levels of ApoB and LDL-C [21]. A working hypothesis has been proposed where by the protein sortilin binds both intracellular apoB100 and extracellular LDL-C particles targeting them for degradation in the lysosome [30]. The minor allele ‘G’ is LDL-C lowering and hence CHD protective in both Pakistani and NPHSII which is in agreement with previous studies [20, 31]. It is reported that each minor allele is associated with 5-8 mg/dl reduction in LDL-C [32, 33]. Although the mean LDL-C values (3.05 mmol/l) in NPHSII non CHD are higher compared to Pakistani non-CHD (2.19 mmol/l), however, the percentage increase associated with this SNP is not different in the two groups (6.65 % in NPHSII non CHD, 7 % in Pakistani controls). It is evident from CHD risk score calculators that 0.14 mmol/l increase in LDL-C is expected to increase CHD risk by 9 %. So although SNP rs599839 has been reported to be the functional variant at this locus [32], the use of rs646776 as a proxy appears to capture well the important variat at this locus.

The effect of SNP rs1042031 appears to be counter intuitive, as the minor allele ‘A’ is reported CHD risk allele but is LDL-C lowering. In an early meta-analysis of published studies [34], the minor allele was associated with increased risk of CHD whereas, in our study it was LDL-C lowering in both cohorts, which is in agreement with the results of a systematic review investigating variants in *APOB* and lipid levels although, this study found no significant association between the rs1042031 and CHD [35]. The major reported GWAS hit for LDL-C levels in *APOB* is the miss sense variant rs1367117, but there is very low LD (r^2^ = 0.01 in the 1000 Genomes pilot 1 CEU data) between this and rs1042031 [25]. The SNP rs1042031 is also in weak LD (r^2^ ~ 0.3 in the 1000 Genomes pilot 1 CEU data) with another GWAS CHD hit rs515135, where the minor allele was found to decrease CHD risk. For this SNP, the mechanism is unclear and is suggested that resulting amino acid change alters the charge which may change the tertiary structure of the protein and reduce the affinity of the LDL-C particle for the LDL-receptor, although no evidence has been published to support this hypothesis so far. There also remains the possibility that rs1042031 does not exert an effect itself, but is tagging a functional SNP elsewhere in the *APOB* gene [36, 37]. The LD between SNPs in associated intervals and differing LD patterns among ethnicities suggests that LDL-C and CHD may not have simple molecular basis in the *APOB* gene [37].

For *APOE* it is clear that the variants used are themselves functional so any major difference in overall effect size on LDL-C levels in UK and Pakistani subjects are likely due to differences in *APOE* frequency. Human apoE, acting as a ligand for LDL-receptor, has role in clearance of VLDL remnants hence decreasing serum total cholesterol [38–40]. It is estimated that inherited factors explain about 60 % in determining the levels of plasma total cholesterol and *APOE* polymorphisms contribute 14 % among this genetically determined portion [41]. The concentration of serum LDL-C in subjects with different *APOE* genotypes is in the order of Ɛ2, Ɛ3 and Ɛ4 and the β effect was significantly higher in CHD patients from the Pakistani population. The proportion of sample variance R^2^ is also significant (*p* < 0.05), in agreement with many other studies carried out in Asians and Americans [4, 16, 42].

Since the effect of SNPs is additive and of modest size, the problem was resolved by a strategy where their effects were combined in a gene score. The similar gene scores of healthy subjects from both populations, and a significant difference in the mean gene score of non-CHD and CHD subjects of both populations indicates that the collective effect of the SNPs under study is similar in both ancestries. The difference in gene score is mainly driven by *SORT1* and *APOB* but they have low weighting, *APOE* has high weighting but the allele frequencies were same between cases and controls. This explains why for unweighted gene score there was a significant different between cases and controls while the weighted score was not.

## Conclusion

In conclusion, the risk alleles of all the SNPs raised LDL-C levels quantitatively in both UK and Pakistani people. The effect size was similar in healthy people of both populations but a bigger effect was observed in Pakistani CHD subjects. The risk allele frequencies of all the 4 SNPs studied were significantly different between Pakistani and UK people and combined gene score of 4 SNPs was significantly associated with CHD risk in both populations.

### Limitations

Comparing the effects of SNPs on LDL-C in healthy subjects from Pakistan and UK may be confounded by different genetic backgrounds and environmental factors. While the frequency of these SNPs was significantly different between the populations, the effect sizes of risk alleles on LDL-C were not, indicating that these concerns are unfounded. There appears a larger variance in the UK than in the Pakistaini subjects, such that the proportion of sample variance explained by the SNPs was much smaller in the UK sample, suggesting a greater heterogeneity, but whether this is genetic or environmental (or both) cannot be resolved using these data. The Pakistani cohort contained both males and females while the UK group was only men, however, the frequency of these autosomal SNPs should not be different in the two sexes, and although effect sizes may be of different magnitude in men and women, such differences are usually minimal. Also the sex heterogeneity in the allele frequencies of the studied SNPs has not been reported in previous meta analysis [25]. To confirm in more detail the relationship between these SNPs and CHD in Pakistan, more studies with bigger sample size are required. As each SNP has a small effect on the outcome, new SNPs are required to be included, to construct a better fit score for such multifactorial disease. The Pakistani population, like the rest of the South Asian subcontinent has, to date, been under represented in genetic studies like Hap Map and 1000 genome projects. This study, therefore adds new data to the field of the genetics of LDL-C levels and CHD risk in this population.

## Additional files

Additional file 1: Table S1. Basic features of SNPs under study. (DOC 33 kb) Additional file 2: Table S2. Comparison of RAFs between Pakistani and NPHSII study groups. (DOC 31 kb) Additional file 3: Figure S1. Box plot showing the distribution of gene score in non CHD and CHD subjects and its association with LDL-C in Pakistani people. The figure shows the distribution of gene score in Pakistani CHD and non CHD. It is clear that the gene score is high in CHD than non CHD and LDL-C levels are also high along high gene score. (DOCX 32 kb) Additional file 4: Table S3. Mean LDL-C by number of LDL-C raising SNPs for the unweighted gene score. (DOC 37 kb)
