# Supplementary material for: Effect of SORT1, APOB and APOE polymorphisms on LDL-C and coronary heart disease in Pakistani subjects and their comparison with Northwick Park Heart Study II
Source: Lipids Health Dis. 2016 Apr 26;15:83. doi: 10.1186/s12944-016-0253-0 (PMC4845441; doi:10.1186/s12944-016-0253-0)
Supplement: Additional file 1: Table S1. — Basic features of SNPs under study. (DOC 33 kb) [file 12944_2016_253_MOESM1_ESM.doc]

Supplementary Table 1: Basic features of SNPs under study.

|  |  |  | Pakistani | | NPHSII | |
| --- | --- | --- | --- | --- | --- | --- |
| CHR | Gene | SNP | Call rate % | HWE-*p*  Non CHD/CHD | Call rate (%) | HWE-*p* non CHD/CHD |
| 1p133 | *APOB* | rs1042031 | 97 | 1.00/0.74 | 99 | 0.16/0.02 |
| 2p23 | *SORT1* | rs646776 | 97 | 0.14/0.59 | 98 | 0.27/0.01 |
| 19q13 | *APOE 112* | rs429358 | 96 | 0.67/0.28 | 97 | 0.66/0.57 |
| 19q13 | *APOE 158* | rs7412 | 96 | 1.00/0.13 | 97 | 0.71/0.88 |

CHR: chromosomal location, HWE-*p*: Hardy Weinberg equilibrium significance level.
