# Supplementary material for: Effect of SORT1, APOB and APOE polymorphisms on LDL-C and coronary heart disease in Pakistani subjects and their comparison with Northwick Park Heart Study II
Source: Lipids Health Dis. 2016 Apr 26;15:83. doi: 10.1186/s12944-016-0253-0 (PMC4845441; doi:10.1186/s12944-016-0253-0)
Supplement: Additional file 2: Table S2. — Comparison of RAFs between Pakistani and NPHSII study groups. (DOC 31 kb) [file 12944_2016_253_MOESM2_ESM.doc]

**Supplementary Table 2: Comparison of RAFs between Pakistani and NPHSII** study groups.

|  |  | RAFs in Pakistani samples | | | RAFs in NPHSII | | |  |  |
| --- | --- | --- | --- | --- | --- | --- | --- | --- | --- |
| SNP | Alleles | Non CHD | CHD | *p* | Non CHD | CHD | *p* | NPHSII non CHD vs. Pakistani non CHD *p-*value | NPHSII CHD vs. Pakistani CHD  *p*-value |
| rs646776 | G/A* | 0.72 | 0.75 | 0.19 | 0.79 | 0.81 | 0.38 | 0.001 | 0.04 |
| rs1042031 | A/G* | 0.87 | 0.92 | 0.007 | 0.81 | 0.83 | 0.39 | 0.002 | 5.9x10^-6^ |
| rs429358 | T/C* | 0.11 | 0.12 | 0.46 | 0.14 | 0.16 | 0.30 | 0.03 | 0.04 |
| rs7412 | C/T* | 0.04 | 0.04 | 0.98 | 0.09 | 0.07 | 0.08 | 0.0002 | 0.05 |

* the risk allele, RAF: risk allele frequency, risk allele means LDL-C raising allele.
