# Supplementary figures and images for: Effect of SORT1, APOB and APOE polymorphisms on LDL-C and coronary heart disease in Pakistani subjects and their comparison with Northwick Park Heart Study II
Source: Lipids Health Dis. 2016 Apr 26;15:83. doi: 10.1186/s12944-016-0253-0 (PMC4845441; doi:10.1186/s12944-016-0253-0)

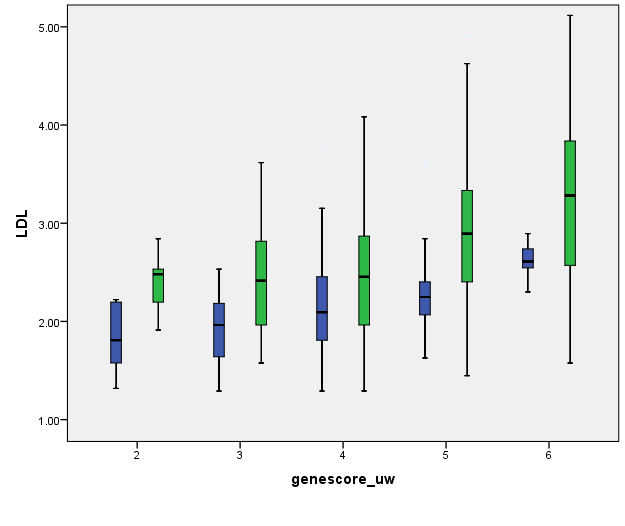

Supplement: Additional file 3: Figure S1. — Box plot showing the distribution of gene score in non CHD and CHD subjects and its association with LDL-C in Pakistani people. The figure shows the distribution of gene score in Pakistani CHD and non CHD. It is clear that the gene score is high in CHD than non CHD and LDL-C levels are also high along high gene score. (DOCX 32 kb) [file 12944_2016_253_MOESM3_ESM.docx]
