# Supplementary material for: Effect of SORT1, APOB and APOE polymorphisms on LDL-C and coronary heart disease in Pakistani subjects and their comparison with Northwick Park Heart Study II
Source: Lipids Health Dis. 2016 Apr 26;15:83. doi: 10.1186/s12944-016-0253-0 (PMC4845441; doi:10.1186/s12944-016-0253-0)
Supplement: Additional file 4: Table S3. — Mean LDL-C by number of LDL-C raising SNPs for the unweighted gene score. (DOC 37 kb) [file 12944_2016_253_MOESM4_ESM.doc]

**Supplementary Table 3: Mean LDL-C by number of LDL-C raising SNPs for the unweighted gene score.**

| Unweighted gene score | NPHSII non CHD | NPHSII CHD | Pakistani non CHD |  | Pakistani CHD |  |
| --- | --- | --- | --- | --- | --- | --- |
| 1 | 2.17 (0.57)  N=10 | - | - |  | - |  |
| 2 | 2.43 (0.92)  N=79 | 3.13 (1.03)  N=6 | 1.81(0.33)  N=9 |  | 2.36 (0.31)  N=9 |  |
| 3 | 2.89 (1.02)  N=287 | 3.05 (0.83)  N=28 | 1.94 (0.35)  N=32 |  | 2.44 (0.54)  N=40 |  |
| 4 | 3.00 (0.99)  N=627 | 3.43 (0.89)  N=63 | 2.18 (0.53)  N=81 |  | 2.50 ( 0.70)  N=159 |  |
| 5 | 3.20 (1.01)  N=596 | 3.38 (1.03)  N=75 | 2.26 (0.30)  N=82 |  | 2.94 (0.72)  N=145 |  |
| 6 | 3.33 (0.97) N=171 | 3.51 (0.84)  N=23 | 2.62 (0.16)  N=15 |  | 3.21 (0.78)  N=51 |  |
| *p-*value (ANOVA) | 1.4x10^-14^ | 0.38 | 3.1x10^-7^ |  | 2.9x10^-12^ |  |
| Δ | 0.9 | 0.38 | 0.81 |  | 0.85 |  |

Δ is the difference in LDL-C between individuals having 2 with those having 6 risk alleles.
